# Evaluation of Field Variation in Flower Thrips, *Frankliniella intonsa* Trybom to Spinosoid Insecticide in Inner Mongolia, China

**DOI:** 10.3390/insects17050511

**Published:** 2026-05-18

**Authors:** Wenxue Bao, Nan Wu, Angeer, Zhaorigetu Hubhachen, Yue Gao

**Affiliations:** 1 College of Forestry, Inner Mongolia Agriculture University, Hohhot 010018, China; aaa18747193892@163.com (N.W.); 18686028571@163.com (A.); yuegao@imau.edu.cn (Y.G.); 2 Agriculture Research and Development Program, Central State University, Wilberforce, OH 45384, USA

**Keywords:** flower thrip, spinosoid, field variation, insecticide susceptibility, CYP450 enzymes, *nAChRα6*, Inner Mongolia

## Abstract

Flower thrips are a major vegetable and crop pest in Inner Mongolia, China. Due to the frequent application of insecticides for pest control, insecticide resistance in the species has been reported previously. Therefore, we evaluated the field variation in four populations due to the use of spinosoid insecticides, spinosad and spinetoram, by assessing stomach and contact toxicities. The LC_50_ value of the stomach toxicity for the DLT population for spinosad and spinetoram was 1.22–2.25 and 1.19–1.90-fold higher, respectively, compared with values in the other three populations. The LC_99_ value of stomach toxicity for the QBM population for spinosad was 9.22–161.57-fold higher compared with the other three populations, whereas the value for spinetoram in the HHG population was 3.28–6.77-fold higher compared with the other three populations. These results clearly showed that the field variation due to spinosoid insecticide use differed across the region. For spinosad, the LC_99_ values for stomach toxicity were higher than those for contact toxicity in all four populations, with action ratios ranging from 0.031 to 0.448. In contrast, for spinetoram, the LC_99_ values for stomach toxicity were lower than those for contact toxicity in all populations, with action ratios ranging from 52.64 to 1901.21. Sequence analysis of the *FIα6* subunit of the *nAChRα6* gene revealed no point mutations in any of the four populations. Therefore, we further investigated the activity of the detoxification enzyme, CYP450s, by analyzing the synergistic effect of piperonyl butoxide (PBO) on the different susceptibilities to spinosad in the populations. The LC_99_ value of spinosad was reduced 59.64-fold in the PBO-treated DLT population and 2.52-fold in the PBO-treated HLG population compared with the untreated population. These results clearly indicate that the field variation in flower thrips due to spinosoid insecticides in Inner Mongolia, China, may be due to the activation of the detoxification enzyme, CYP450s.

## Simple Summary

This study reports on the field variation in the flower thrips, *Frankliniella intonsa*, based on spinosoid insecticide use in Inner Mongolia, China. Our results show that the field-collected populations of the species had different values of LC_50_ and LC_99_, indicating reduced susceptibility to spinosoid insecticides. However, there was no point mutation in the FIα6 subunit of the *nAChRα6* gene. The field variation in flower thrips due to spinosoid insecticide use in Inner Mongolia, China, may be due to the activation of the detoxification enzyme, CYP450s.

## 1. Introduction

In China, the flower thrips, *Frankliniella intonsa* Trybom (Thripidae, Thysanoptera), is a major pest for crops, forages, and flora [1,2,3]. Strawberry, *Fragaria ananassa* Duch, and alfalfa, *Medicago sativa* L., are the main hosts for the species [4]. The pest population increases in June each year and causes serious reductions in alfalfa yield from late August to early September [4]. With the increased planting of alfalfa in Inner Mongolia, China, in recent years, serious economic losses have been reported due to pest damage. Similar to other regions in Inner Mongolia, China, insecticide application is the main approach for controlling the pest. However, frequent and intensive insecticide use has led to reduced susceptibility in field populations, as reported by local growers (personal communication). Most recently, susceptibility of field populations of *F. intonsa* to spinetoram, imidacloprid, and acetamiprid in Xinjiang cotton fields, China [5]. Since 2019, we have evaluated the field variation in *F. intonsa* based on several insecticide classes, including pyrethroids, neonicotinoids, avermectins, and spinosoids. In this paper, we report on the field variation in the populations based on the spinosoid insecticides, spinosad and spinetoram, assessed through stomach and contact toxicity bioassays.

It is well known that spinosoid insecticides act on the nicotinic acetylcholine receptor (nAChR) on the postsynaptic membrane of the nervous system. Spinosoid insecticides cause changes in the configuration of nAChR, leading to muscle spasms and, finally, the death of the pest due to paralysis [6]. Insects’ nAChRs consist of two α and three β subunits. Previous studies have shown that resistance to spinosoids is closely associated with mutations in the α6 subunit of nAChR. For example, the loss of a partial sequence in the α6 subunit of nAChR after knockout in the fruit fly, *Drosophila melanogaster*, caused high resistance to spinosad [7]. Spinosad resistance in the oriental fruit fly, *Bactrocera dorsalis*, and cabbage moth, *Plutella xylostella*, is due to a point mutation in the α6 subunit of nAChR, which leads to mis-splicing and the production of a new stop codon [8,9]. The mutation of the amino acid at position 275 (G275E) in α6 of the nAChR enzyme in western flower thrips, *Frankliniella occidentalia*, and melon thrips, *Thrips palmi*, confers spinosad resistance in these species [10,11,12]. In addition, the mutation of the amino acid at position 275 (G275V) in α6 of the nAChR enzyme in western flower thrips, *F. occidentalia*, was shown to lead to the reduced susceptibility to spinosad in a laboratory-selected population of the species in Japan [13]. Therefore, we sequenced and analyzed the α6 subunit of the *nAChRα6* gene to confirm whether the field variation based on spinosad use in field-collected populations from Inner Mongolia, China, is related to the mutation at position 275 of the enzyme.

In addition to target site mutations, resistance to spinosad in several insect pests—including the beet armyworm, *Spodoptera exigua*; cotton bollworm, *Helicoverpa armigera*; cotton leafworm, *Spodoptera litura*; and melon thrips, *T. palmi*—has been linked to the elevated detoxification activity of cytochrome P450 monooxygenases (CYP450s) [12,14,15,16]. Therefore, we also examined CYP450 activity in field populations of *F. intonsa* from Inner Mongolia by assessing the synergistic effects of piperonyl butoxide (PBO), a known P450 inhibitor.

## 2. Materials and Methods

### 2.1. Biological Samples

Four populations, namely DLT, HHG, QBM, and HLG, were collected from *Medicago sativa* from Dalad Banner (E 110°16′47″, N 40°26′31″), Chahar Right Wing Banner (E 112°32′03″, N 41°08′17″), Hohhot city (E 111°48′01″, N 40°43′31″) and Horinger Banner (E 111°30′16″, N 40°23′34″), Inner Mongolia, China, during the summer of 2020. Then, the populations (F0) were transported to the laboratory at Inner Mongolian Agricultural University and reared on faba bean in an environmental chamber at 25 °C, 65 ± 5% RH, with 16L:8D. One- to two-week-old thrips from generations F30–F35 were used in the experiment in 2022.

### 2.2. Insecticides

Spinosad 3% EW was obtained from Haite Chemical Manufacturing LLC (Tianmen, Hubei, China); spinetoram 60 g/L SC was purchased from Corteva Agriscience, Shanghai, China; and PBO was obtained from Hebei Bailingwei Super Fine Materials LLC (Shijiazhuang, China ). NAiso Plus for the extraction of Total RNA, PrimeScript™ II 1st Strand cDNA Synthesis Kit, Recombinant DNase I, RNase-free, Premix Taq™ (TaKaRa Taq™ Version 2.0 plus dye), and TaKaRa MiniBEST Plasmid Purification Kit Ver.4.0 were purchased from Takara Bio Inc., Beijing, China.

### 2.3. Bioassay for Stomach Toxicity

The stomach toxicity of spinosoid insecticides to flower thrips was evaluated using the leaf dipping method described by Falmy et al. [17]. Each insecticide was serially diluted with acetone (99.9%) to obtain 5 different concentrations, which provided <5% to 100% mortality of female flower thrips. Faba bean, *Vicia faba*, leaves (about 3.5 cm × 2 cm in size) were washed with distilled water with 0.1% spreading agent and then dipped in the different concentrations of insecticides for 30 sec. Leaves dipped in the distilled water with 0.1% spreading agent were treated as controls. Treated leaves were air-dried on paper towels at room temperature and then placed in Petri dishes (3.8 cm diameter × 1.0 cm height). A total of 15–30 female adults were introduced into each Petri dish, which were sealed using parafilm. Then, the samples were held in an environmental chamber for 48 h at 25 °C and 65 ± 5% RH, with a 16L:8D photoperiod. Each individual thrip was gently touched with a fine paintbrush to determine whether it moved (alive) or did not move (dead). A bioassay was conducted with at least three replicates. Corrected mortality was calculated using Abbott’s formula [18].

### 2.4. Bioassay for Contact Toxicity

Five serial concentrations of each insecticide were prepared as described earlier. For each concentration, 70 µL of solution was transferred into a 2 mL scintillation vial (315 mm × 12 mm) and evenly coated on the inner wall by rolling at 75 rpm on a roller (JL GUIII, Shanghai Jinlan Instrument Manufacturing Co., Ltd., Shanghai, China) until the acetone evaporated completely. A total of 15–30 female adults were released into each vial, which was capped with a lid containing an opening covered by a fine mesh. All subsequent procedures were identical to those described for stomach toxicity, except that mortality was recorded after 8 h [19].

### 2.5. Synergism Test

The synergistic effect of PBO with the insecticides was tested using the leaf dipping method mentioned earlier and described by Fahmy et al. [17]. PBO was diluted with acetone and then combined with the insecticides, which contained 0.1% spreading agent. The final content of acetone in each vial was 0.1%, while the final concentrations of PBO were 0.295 µL L^−1^ [20]. The treatment of leaves and the following experimental procedures were the same as those described in Section 2.3.

### 2.6. Total RNA Extraction and RT-PCR

Total RNA from 100 female adults with three replicates from each population was extracted using the NAiso Plus Kit (Takara Bio Inc., Beijing, China 102206). The reverse transcription reaction was conducted using an Oligo dT Primer (Takara) PrimeScript™ II 1st Strand cDNA Synthesis Kit and Recombinant DNase I, with 500 ng total RNA used as the template. Then, 5′-end and 3′-end cDNA were synthesized using 5′-RACE and 3′-RACE kits. The 5′-end cDNA was synthesized using the pair of primers, RC970-C-RT1 (CACCATATTGAGGAACACAGTCAAC)/RC970-C-RT2 (GTGGCAGAGTGAACCCGAGTAG). The full-length cDNA of the nAChRα6 subunit was amplified using Premix Taq™. Primers were designed based on the α6 subunit sequence of occidentalis (GenBank/EMBL/DDBJ accession number: KU557780; Table 1).

The *nAChRα6* subunit from the four field-collected populations was connected with the vector, pMD18-T, and cloned into the competent cell, SK2301. Then, the cloned fragments from 5 clones in each population were amplified using the universal primers (M13+(−47): AGGGTTTTCCCAGTCACG and M13−(−48): GAGCGGATAACAATTTCACAC). The PCR temperature program was as follows: denaturation at 95 °C for 3 min; 25 cycles at 94 °C for 15 s, 55 °C for 30 s, and 72 °C for 2 min for denaturation, annealing, and extension, respectively; and a final extension at 72 °C for 7 min.

### 2.7. Sequencing

After they were purified using a TaKaRa MiniBEST Plasmid Purification Kit Ver. 4.0 (Takara Bio Inc., Beijing, China), the purified plasmid DNAs were sequenced directly at Beijing Genomics Institute (Beijing, China). The primers used for gene amplification are shown in Table 1. Nucleotide and deduced amino acid sequences were analyzed using DNAMAN V9.0. Nucleotide sequences and deduced amino acid sequences from flower thrips were aligned against Western flower thrip and melon thrip populations using Clustal W2 (http://www.genome.jp/tools/clustalw/, accessed on 19 December 2023).

### 2.8. Data Analysis

The insecticide concentrations that caused 50, 90, and 99 percent mortality of thrips (LC50, LC90, and LC99) were determined for each insecticide for the field-collected populations using probit analysis with goodness-of-fit tests on the data after Abbott’s correction for control mortality [18,21,22].

## 3. Results

### 3.1. Field Variation in Flower Thrip Stomach Toxicity Based on Spinosoid Insecticide Use

The susceptibility of *F. intonsa* to spinosoid insecticides varied between the four field-collected populations. The LC_50_ values of spinosad for stomach toxicity in the DL, HHG, QBM, and HLG populations were 0.099, 0.045, 0.081, and 0.044 µL L^−1^, respectively, whereas the LC_99_ values in the same populations were 24.454, 103.362, 952.765, and 5.897 µL L^−1^, respectively. The LC_50_ value of the DLT population was 1.22–2.25-fold higher compared with that in the other three populations, whereas the LC_99_ value of the QBM population was 9.22–161.57-fold higher compared with that in the other three populations (Table 2).

The LC_50_ values of spinetoram for stomach toxicity in the DLT, HHG, QBM, and HLG populations were 0.019, 0.01, 0.016, and 0.01 µL L^−1^, respectively, whereas the LC_99_ values of spinetoram for stomach toxicity in the DLT, HHG, QBM, and HLG populations were 0.096, 0.528, 0.078, and 0.161 µL L^−1^, respectively. The LC_50_ value of the DLT population was 1.19–1.90-fold higher compared with that in the other three populations, whereas the LC_99_ value of the HHG population was 3.28–6.77-fold higher compared with that in the other three populations (Table 3).

The susceptibility of the flower thrips to spinosoid insecticides based on contact toxicity also varied across the different collection sites. The LC_50_ values of spinosad for contact toxicity in the DLT, HHG, QBM, and HLG populations were 0.199, 0.347, 0.329, and 0.192 µL L^−1^, respectively, whereas the LC_99_ values in the same populations were 4.196, 4.338, 29.233, and 2.642 µL L^−1^, respectively (Table 2). The LC_50_ values of spinetoram for contact toxicity in the DLT, HHG, QBM, and HLG populations were 0.238, 0.134, 0.478, and 0.104 µL L^−1^, respectively, whereas the LC_99_ values of spinetoram for stomach toxicity in the DLT, HHG, QBM, and HLG populations were 5.053, 83.865, 72.86, and 306.094 µL L^−1^, respectively. The LC_50_ value of the QBM population was 2.008–4.596-fold higher compared with that in the other three populations, whereas the LC_99_ value of the HLG population was 3.650–60.577-fold higher compared with that in the other three populations (Table 3). The action ratios of the LC_50_ of spinosad in the DLT, HHG, QBM, and HLG populations were 2.010, 7.711, 4.062, and 4.136, respectively, whereas those of LC_99_ in the same populations were 0.172, 0.042, 0.031, and 0.448, respectively (Table 3). The action ratios of the LC_50_ of spinetoram in the DLT, HHG, QBM, and HLG populations were 12.526, 13.400, 29.875, and 10.400, respectively, whereas the LC_99_ values for the same populations were 52.632, 158.835, 934.103, and 1901.205, respectively (Table 3).

### 3.2. The Gene Sequence of nAChRα6 Subunit

**Figure 1:**
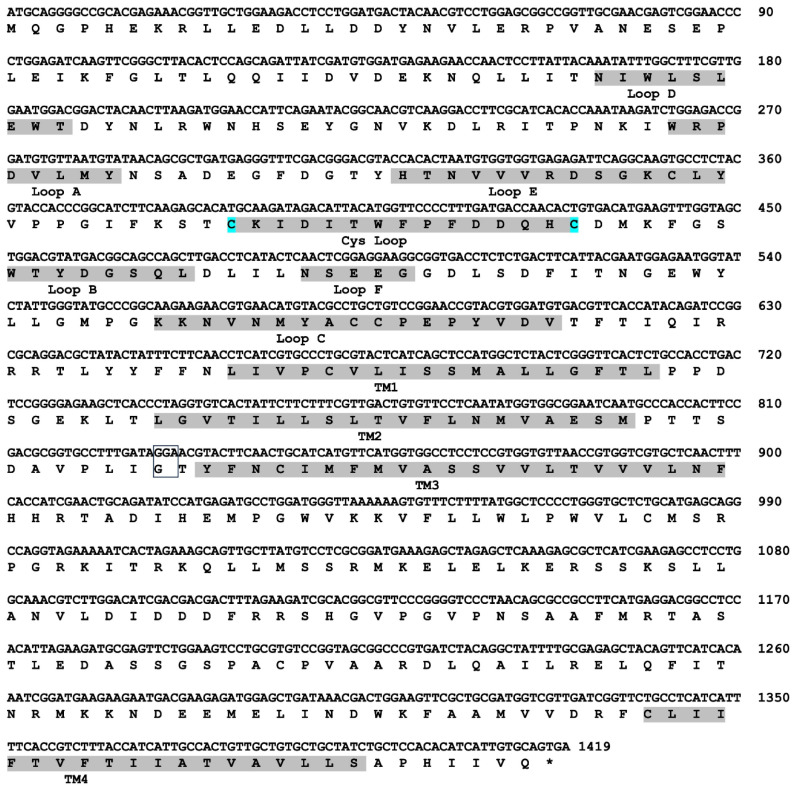
The nucleotide and the deduced amino acid sequences of the FIα6 subunit of the flower thrips, *F. intonsa.* The membrane-spanning domain FIα6 (TM1, TM2, TM3, TM4), the Cys loop (cysteines at the two ends shown in blue) and the 6 loops (Loops D, A, E, B, F, C), which are located outside the membrane of the N-end of FIα6, are shown in the shaded areas. The position of amino acid #275 and its corresponding nucleotides are shown as a rectangle.

The full-length nucleotide sequence of the *nAChRα6* subunit in *F. intonsa* was 1416 bp, encoding a predicted protein with 472 amino acids (accession no. PZ142668) (Figure 1). The deduced amino acid sequence showed high similarity with those of other thrips species, with 99.79% similarity to *F. occidentalis* and 96.84% similarity to *T. palmi* (accession nos. BAN81845 and BAP18687). The whole sequence we obtained belongs to the FIα6 subunit of nAchRα6, in which we identified two selective exons, 8a and 3b, from the selective exons, 8a/8b and 3a/3b, of the *nAchRα6* subunit in *F. occidentalis* and *T. palmi*.

**Figure 2:**
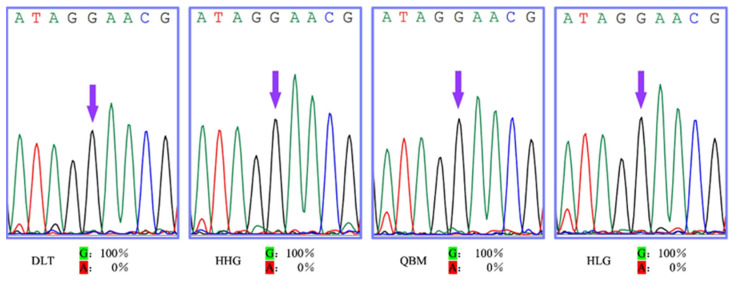
The partial sequences of the subunit (*α6*) of the *nAChR* gene from the flower thrip, *F. intonsa*. The arrow heads show the point mutation site in the gene. No mutation was observed in the 4 field-collected populations of the species from Inner Mongolia, China.

Furthermore, we analyzed the deduced amino acid sequences of the FIα6 subunit of the four populations and found that there were no point mutations like the one amino acid mutation at position 275 in other thrips (Figure 2).

### 3.3. Synergistic Effect of PBO on Detoxification Enzyme, CYP450

The LC_50_ and LC_99_ values of spinosad in the PBO-treated DLT population showed 1.33- and 59.64-fold decreases compared with those in the untreated population. The LC_50_ value in the PBO-treated HLG population increased 5.21-fold, whereas the LC_99_ value in the population decreased 2.52-fold compared with that in the untreated population.

## 4. Discussion

Here, we report on the field variations in four field-collected populations of flower thrips based on spinosoid insecticide use, including spinosad and spinetoram, in Inner Mongolia, China. To the best of our knowledge, this is the first report on this particular topic in the region. Our results showed that the susceptibility of the populations to these insecticides varied in stomach and contact toxicity bioassays.

We found that the LC_50_ value of the DLT population in the stomach toxicity bioassay was 1.22–2.25-fold higher compared with that in the other three populations, whereas the LC_99_ value of the QBM population was 9.22–161.57-fold higher compared with that in the other three populations in the spinosad insecticide bioassay (Table 2). Furthermore, we discovered that the LC_50_ value of spinetoram for stomach toxicity in the DLT population was 1.19–1.90-fold higher compared with that in the other three populations, whereas the LC_99_ value of spinetoram for stomach toxicity in the HHG population was 3.28–6.77-fold higher compared with that in the other three populations (Table 3). These results clearly indicate that the pest’s response to spinosoid insecticides varied across the field-collected populations collected in Inner Mongolia, China.

We found that the LC_50_ values for the stomach toxicity of spinosad in the DLT, HHG, QBM, and HLG populations were 50.51–113.64-fold lower than in the susceptible population of *F. intonsa* Trybom [13]. This indicated that all four populations of the species from Inner Mongolia, China, were susceptible to spinosad insecticide based on our current experimental conditions. However, we found that there was a significant difference in the LC_99_ values of the insecticides in these populations. For example, the LC_99_ value was 161.57-fold higher in the QBM population compared with that in the HLG population (Table 2).

We also found that the stomach toxicity of spinosad was higher than its contact toxicity in the populations, whereas that of spinetoram was lower than its contact toxicity when we compared the LC_99_ value of the insecticide across the populations (Table 2 and Table 3).

The LC_50_ value for the stomach toxicity of spinetoram in the four field-collected populations was 4.40–5.2-fold higher than that of spinosad, whereas the LC_50_ value for the contact toxicity of spinetoram was 0.688–2.589-fold higher than that of spinosad. Our results are consistent with the findings of Crouse et al. [23], who described that the toxicity of spinetoram was higher than that of spinosad. Consistent with the finding in the flower thrips, *F. intonsa*, in the cotton field in Xinjiang, China, by Li et al. [5], field variation to spinetoram as well as spinosad was observed in the pest in Inner Mongolia, China.

Our results clearly show that the response to the insecticides differs across the field-collected populations of the flower thrip, *F. intonsa*, collected in Inner Mongolia, China. Therefore, we further investigated whether the varying susceptibility to the spinosoid insecticides in the populations was due to a gene mutation in the subunit of the gene, nAChRα6. Previous studies [8,9,24] have shown that spinosad resistance in the oriental fruit fly, *B. dorsalis*, and the cabbage moth, *P. xylostella*, was due to a point mutation in the subunit *α6* of the *nAChR* gene, which produced mis-splicing and a new stop codon in the resistant populations. However, based on *nAChR* gene sequencing across the field-collected populations of the species in the regions, we did not find any mutations (Figure 2). Our results indicate that the varying response to the spinosoid insecticides in these populations was not related to a gene mutation in the subunit of the *nAChR* gene. Therefore, we further investigated the activity of the detoxification enzyme, CYP450, in the field-collected populations. Researchers have observed spinosad resistance due to increased detoxification by the enzyme CYP450 in *F. occidentalis* [11], *S. exigua* [14], *H. armigera* [15], and *S. litura* [16]. We treated thrips from the DLT and HLG populations with spinosad insecticide with or without PBO, which is a CYP450 enzyme inhibitor. We discovered that LC_50_ and LC_99_ values in the thrips from the DLT population decreased 1.33- and 59.64-fold, respectively, compared with those in the thrips without PBO (Table 4). It was surprising to find that the LC_50_ value in the HLG population increased 5.21-fold, whereas the LC_99_ value decreased 2.52-fold compared with those in the thrips without PBO (Table 4). Contrary to the expected result, the LC_50_ value in the HLG population increased in the PBO-treated group. We will evaluate the CYP450 enzyme activity directly using more populations of the species treated with spinosad and spinetoram insecticides in a future study. Taken together, our results show that the field variation in these populations may be due to the activation of the detoxification enzyme, CYP450s [13]. In conclusion, our results indicate that the response to spinosoid insecticides in these populations of flower thrips differed across the regions. However, due to the lack of a susceptible population for control, the field variation of the insecticides in the pest could be treated as the comparative susceptibility in the species. These differences may be due to the activation of the detoxification enzyme, CYP450s, instead of the gene mutation in the subunit of the *nAChR* gene. A recent study described that the resistant allele frequency of spinetoram in *Drosophila melanogaster* in vineyards in New York, USA, increased over time [25]. Therefore, to overcome the limitations in sample size, collection regions, and dose points in the probit analysis, we will collect more samples of the species from additional areas in Inner Mongolia and northern China to investigate and establish a solid susceptible population. We aim to create a clear picture of spinosoid insecticide resistance and investigate other insecticide families as well within the species through future research.

## Figures and Tables

**Table 1:** Primers used for the amplification of the nAChRα6 subunit in the flower thrips, *F. intonsa*.

Fragments	5′-Primers		3′-Primers	
	Primer Name	Sequence	Primer Name	Sequence
5′RACE	1st 5′adaptor	5′-GCTGTCAACGATACGCTACGTAACG GCATGACAGTG-3′	RC970-C-R5	5′-CCTCCGAGTTGAGTATGAGGTCAAG-3′
	2nd 5.3′outer	5′-GCTGTCAACGATACGCTACGTAAC-3′	RC970-C-R6	5′-CGTCCAGCTACCAAACTTCATGTCA-3′
1	FInAchR_alpha6/2-1F	5′-GCGGCCATCTTAACGACGAT-3′	FInAchR_alpha6/2-1R	5′-GGACAGCAGGCGTACATGTT-3′
2	FInAchR_alpha6/2-2F	5′-GCAGCCAGCTGGACCTCATT-3′	FInAchR_alpha6/2-2R	5′-TGTGTGGAGCTGATAGCAGC-3′
3′RACE	1st RC970-C-F1	5′-CGTCTTGGACATCGACGACG-3′	5.3′outer	5′-GCTGTCAACGATACGCTACGTAAC-3′
	2nd RC970-C-F2	5′-TAACAGCGCCGCCTTCATGA-3′	5.3′inner	5′-GCTACGTAACGGCATGACAGTG-3′

**Table 2:** The field variation in spinosad in the flower thrips, *F. intonsa.*

Population	Treatment	*n*	LC_50_ (95% CL)	LC_90_ (95% CL)	LC_99_ (95% CL)	Slope	Y Intercept	X^2^ (df)	*p*-Value
DLT	ST	366 (117, 123, 126)	0.099 (0.012–0.446)	1.378 (0.338–610.535)	24.454 (2.294–9,074,182.950)	1.92	1.93	17.429 (4)	0.002
	CT	357 (115, 122, 120)	0.199 (0.14–0.276)	0.854 (0.563–1.633)	4.196 (2.069–14.092)	3.468	2.435	4.249 (3)	0.236
	AR		2.010	0.620	0.172				
HHG	ST	336 (107, 126, 103)	0.045 (0.025–0.077)	1.815 (0.738–8.209)	103.362 (18.391–2192.647)	1.366	1.844	6.402 (4)	0.171
	CT	401 (138, 144, 119)	0.347 (0.251–0.48)	1.161 (0.793–2.021)	4.338 (2.402–11.215)	4.189	1.926	0.2 (3)	0.978
	AR		7.711	0.640	0.042				
QBM	ST	315 (105, 89, 121)	0.081 (0.003–21.919)	7.156 (0.513–4.066 × 10^20^)	952.765 (8.136–8.129 × 10^42^)	1.129	1.232	16.256 (4)	0.003
	CT	517 (192, 160, 165)	0.329 (0.232–0.456)	2.813 (1.811–5.173)	29.233 (13.365–93.396)	2.359	1.138	3.948 (3)	0.267
	AR		4.062	0.393	0.031				
HLG	ST	362 (103, 120, 139)	0.044 (0.001–0.246)	0.458 (0.113–11,633.034)	5.897 (0.608–3.782 × 10^10^)	2.161	2.93	21.9 (4)	0
	CT	391 (109, 141, 141)	0.182 (0.034–1.072)	0.654 (0.256–2476.403)	2.642 (0.644–41,915,086.75)	3.953	2.928	11.024 (3)	0.012
	AR		4.136	1.428	0.448				

CL: confidence limit; ST: stomach toxicity; CT: contact toxicity; AR (action ratio): LC_50_ of contact action/LC_50_ of stomach action. “*n*” represents the total number of thrips used with 5 concentrations of spinosad and 3 replicates in the experiment.

**Table 3:** The field variation in spinetoram in the flower thrips, *F. intonsa*.

Population	Treatment	*n*	LC_50_ (95% CL)	LC_90_ (95% CL)	LC_99_ (95% CL)	Slope	Y Intercept	X^2^ (df)	*p*-Value
DLT	ST	329 (112, 112, 105)	0.019 (0.016–0.022)	0.041 (0.033–0.059)	0.096 (0.066–0.187)	6.521	11.227	3.148 (5)	0.677
	CT	284 (75, 107, 102)	0.238 (0.164–0.362)	1.026 (0.61–2.538)	5.053 (2.146–25.28)	3.464	2.158	0.321 (3)	0.956
	AR		12.526	25.024	52.635				
HHG	ST	418 (131, 147, 140)	0.01 (0.001–0.019)	0.068 (0.036–1.204)	0.528 (0.133–1163.26)	2.7	5.344	13.687 (5)	0.018
	CT	395 (124, 135, 136)	0.134 (0.002–0.521)	2.912 (0.698–8355.035)	83.865 (6.205–2.371 × 10^10^)	1.643	1.435	9.245 (3)	0.026
	AR		13.400	42.824	158.835				
QBM	ST	324 (113, 108, 103)	0.016 (0.014–0.019)	0.034 (0.028–0.05)	0.078 (0.053–0.16)	12.026	12.026	3.17 (5)	0.674
	CT	391 (113, 157, 121)	0.478 (0.263–0.738)	5.286 (3.246–10.953)	72.86 (28.025–373.488)	2.105	0.675	3.802 (3)	0.284
	AR		29.875	155.471	934.103				
HLG	ST	523 (167, 194, 162)	0.01 (0.004–0.015)	0.039 (0.025–0.18)	0.161 (0.063–6.95)	3.869	7.668	12.992 (5)	0.023
	CT	448 (178, 138, 132)	0.104 (0.000–0.605)	4.74 (0.017–5.322)	306.094 (14.4–2.068 × 10^12^)	1.325	1.302	10.512 (3)	0.015
	AR		10.400	121.538	1901.205				

CL: confidence limit; ST: stomach toxicity; CT: contact toxicity; AR (action ratio): LC_50_ of contact action/LC_50_ of stomach action. “*n*” represents the total number of thrips used with 5 concentrations of spinetoram and 3 replicates in the experiment.

**Table 4:** The synergistic effect of butoxide piperonyl on the CYP450 enzyme in the spinosad-treated thrips, *F. intonsa.*

Population	Insecticide	*n*	LC_50_ (95% CL)	LC_90_ (95% CL)	LC_99_ (95% CL)	Slope	Y Intercept	X^2^ (df)	*p*-Value
DLT	Spinosad	366 (117, 123, 126)	0.099 (0.012–0.446)	1.378 (0.338–610.535)	24.454 (2.294–9,074,182.950)	1.92	1.93	17.429(4)	0.002
	Spinosad + PBO	379 (133, 116, 130)	0.073 (0.000–0.099)	0.166 (0.126–2067.139)	0.41 (0.216–1.67 × 10^14^)	6.114	6.962	5.249(4)	0.263
HLG	Spinosad	362 (103, 120, 139)	0.044 (0.001–0.246)	0.458 (0.113–11,633.034)	5.897 (0.608–3.782 × 10^10^)	2.161	2.93	21.9(4)	0
	Spinosad + PBO	501 (189, 162, 150)	0.229 (0.031–0.468)	0.696 (0.363–26.621)	2.338 (0.845–13,871.467)	4.555	2.915	9.921(4)	0.042

PBO: piperonyl butoxide; CL: confidence limit. “*n*” represents the total number of thrips used with 5 concentrations of spinosad and 3 replicates in the experiment.

## Data Availability

The original data are included in the paper. Further inquiries can be directed to the corresponding authors.
